# Maximizing the *Neisseria gonorrhoeae* confirmatory rate and the genotypic detection of ciprofloxacin resistance for samples screened with cobas CT/NG

**DOI:** 10.1128/jcm.01039-23

**Published:** 2023-12-12

**Authors:** Todd M. Pryce, Olivia R. Foti, Erin J. Haygarth, David M. Whiley

**Affiliations:** 1 Department of Clinical Microbiology, PathWest Laboratory Medicine WA, Fiona Stanley Hospital, Murdoch, Western Australia, Australia; 2 Centre for Clinical Research, The University of Queensland, Brisbane, Queensland, Australia; Cleveland Clinic, Cleveland, Ohio, USA

**Keywords:** *Neisseria gonorrhoeae*, confirmatory testing, *gyrA*, ciprofloxacin susceptibility

## Abstract

Supplementary nucleic acid amplification testing for *Neisseria gonorrhoeae* (NG) is widely used to circumvent specificity problems associated with extragenital sites. Here, we compared different supplementary approaches for confirming NG-positive samples from the cobas 4800 CT/NG (c4800) and cobas 6800 CT/NG (c6800) assays using the *ResistancePlus*GC (RP-GC) assay, which in addition to detecting NG, also predicts ciprofloxacin susceptibility via NG *gyrA* characterization. Two different nucleic acid extraction techniques were investigated for RP-GC detection; extracts from c4800 (c4800-RP-GC) and MagNA Pure 96 (MP96-RP-GC). NG-positive (*n* = 300) and -negative (*n* = 150) samples in cobas PCR media from routine c4800 testing were retrospectively retested with c4800, c6800, c4800-RP-GC, and MP96-RP-GC. Selected samples were also tested with Xpert CT/NG (Xpert) for discrepant analysis. The *gyrA* status was compared to ETEST ciprofloxacin susceptibility or non-susceptibility for recovered isolates (*n* = 63). Extragenital confirmatory rates were higher for MP96-RP-GC (131/140; 93.6%) compared to c4800-RP-GC (126/146; 86.3%), albeit not significantly (*P* = 0.6677). Of 9 samples testing positive by c6800 and negative by MP96-RP-GC, 7/9 (77.8%) were also negative by Xpert. By contrast, the number of samples returning a valid *gyrA* status was significantly (*P* = 0.0003) higher for MP96-RP-GC (270/293; 92.2%) compared to c4800-RP-GC (245/298; 82.2%). The overall MP96-RP-GC *gyrA* status correlated 98.4% (61/62) with the reported ciprofloxacin sensitive (35/36; 97.2%) or non-susceptible (26/26; 100%) phenotype. Improved RP-GC confirmatory rates and reported *gyrA* status were observed using MP96 nucleic acids compared to c4800 extracts. The data further highlight the ongoing need for NG supplemental testing for oropharyngeal samples.

## INTRODUCTION

Sexually transmitted infections caused by *Neisseria gonorrhoeae* (NG) are estimated to account for 87 million new infections per year (1). Transmission by direct mucosal contact can lead to asymptomatic or symptomatic infections in the urethra, endocervix, rectum, and pharynx. Nucleic acid amplification tests (NAATs) have largely replaced culture as the primary method for gonorrhea diagnosis (2). Following the implementation of NAATs for NG screening, specificity problems associated with cross-reaction with commensal *Neisseria* species have widely been reported. The problem has been most pronounced in testing oropharyngeal swabs, where commensal *Neisseria* species are ubiquitous (3–5). Earlier generation commercial assays lacked specific claims for testing oropharyngeal samples; however, third-generation commercial NG-NAATs have progressed to include performance claims for extragenital sites, such as oropharyngeal and anorectal swabs (2, 6, 7).

Supplementary testing (whereby samples testing positive in a screening NG-NAAT are confirmed by a second NAAT) has been widely implemented by laboratories to address the issues of non-specificity (8–10). Our laboratory recently evaluated two versions of the cobas CT/NG assay (Roche Molecular Systems Inc., Branchburg, NJ, USA) using the cobas 4800 (c4800) and cobas 6800 (c6800) testing platforms. We showed that supplemental confirmatory testing may not be required for urogenital sites but is warranted for oropharyngeal samples for both assays owing to suboptimal confirmation rates (7). Another recent c6800 study has shown suboptimal confirmatory rates for oropharyngeal samples and advocated secondary testing for oropharyngeal samples in line with the UK guidelines (11).

NG infection is frequently treated empirically upon clinical presentation; however, the efficacy of antimicrobial therapy is threatened by the development of successive NG antimicrobial resistance (AMR) (12, 13). Given the decline in culture and the subsequent reduction of antimicrobial susceptibility data (14), the integration of reliable genotypic AMR markers into NG NAATs allows more appropriate and personalized therapy to combat the growing state of AMR (15). Currently, the prediction of AMR based on genetic changes is most accurate for fluoroquinolones (15–19). Codon 91 gyrase A (*gyrA*) testing has shown to be a reliable predictor of ciprofloxacin resistance in NG and tests targeting this marker may reduce the use of ceftriaxone (20). Considering the pharynx has been suggested as an important site for AMR development due to non-gonococcal *Neisseria* species at the pharyngeal mucosa (21), a dual-purpose NG supplemental test that includes AMR markers would be ideal, particularly for oropharyngeal sites where specificity issues have been a concern.

This study was prompted by our previous concerns relating to suboptimal oropharyngeal confirmatory rates with an in-house supplemental assay detecting *opa* and *porA* (7). The *ResistancePlus*GC assay (RP-GC) (SpeeDx, Eveleigh, NSW, Australia) is a commercial supplemental test that simultaneously detects NG (dual target detection of *opa* and *porA*), the *gyrA* 91S (wild type), or the *gyrA* 91F mutation associated with resistance to ciprofloxacin. This test can be performed from nucleic acid recovered from the cobas 4800 (c4800-RP-GC) or Roche MagNA Pure 96 (MP96-RP-GC) nucleic acids following the methods issued by the manufacturer. Given RP-GC shares the same targets as our in-house assay and co-detects an AMR target, we aimed to assess the suitability for NG confirmation in our population. However, the challenge with NG confirmation following c6800 screening compared to c4800 is the lack of accessible DNA extracts from the c6800 system. Hence, we also sought to compare the MP96 and c4800 extraction methods for RP-GC detection for all samples preserved in cobas PCR media. Xpert CT/NG (Xpert) (Cepheid, Sunnyvale, CA, USA) testing was selectively applied to samples to better understand confirmation data and bacterial culture results were used to assess RP-GC *gyrA* results.

## MATERIALS AND METHODS

### Study overview and sample testing

**Fig 1:**
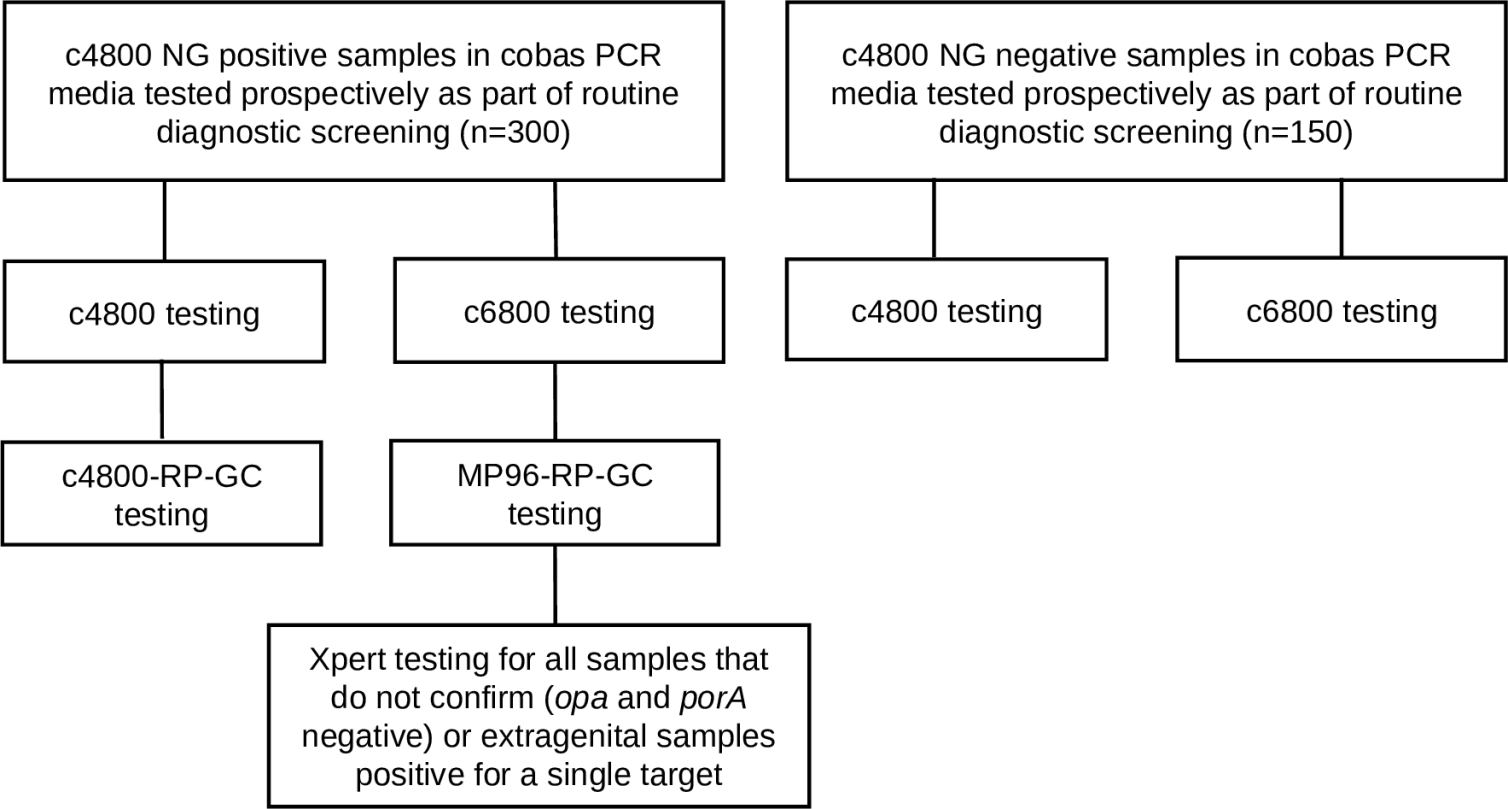
Study overview of the positive and negative samples tested with c4800, c6800, c4800-RP-GC, M96-RP-GC, and Xpert.

A study overview is presented in Fig. 1. Consecutive NG-positive (*n* = 300) and -negative (*n* = 150) samples preserved in cobas PCR media (Roche) were collected in the years 2021–2022, primarily from two large specialist sexual health clinics in Perth, Western Australia. The samples were routinely tested using c4800 following the instructions issued by the manufacturer (Roche) and were selected for this study based on these routine testing results. Samples were stored at room temperature in accordance with the instructions issued by the manufacturer. For this study, all samples were retrospectively retested in a blinded fashion with c4800 and c6800. RP-GC was performed from nucleic acids recovered from c4800 and from MP96 according to the methods issued by the manufacturer (SpeeDx). Briefly, 200 µL of the sample was extracted with MP96 using the MagNA Pure 96 DNA and Viral NA Small Volume Kit and the Pathogen Universal 200 protocol (Roche). An elution volume of 50 µL was used. A volume of 5 µL of either c4800 or MP96 nucleic acids was added to 15 µL of RP-GC master mix. Amplification and detection were performed using the LightCycler 480 (II) instrument using the PCR cycling conditions issued by the manufacturer (SpeeDx). Result analysis was performed using RP-GC (LC480) software version 1.0 with no modifications to target calling. The final NG supplemental result (confirmed or not confirmed) was interpreted according to the NG supplemental testing algorithm described below. Xpert was performed using 1 mL of cobas PCR media on selected samples tested with c6800 according to the NG supplemental testing algorithm below. We note that cobas PCR media has not been validated by the manufacturer for Xpert. However, for consistency, we used the same sample volume of cobas PCR media as stated for the manufacturer’s recommended Xpert urine and vaginal/endocervical collection devices. All retesting was performed on two separate days with a single amplification lot number for each respective assay (c4800, c6800, RP-GC, and Xpert), with RP-GC supplemental testing performed from stored nucleic acids (4°C) within 24 hours of retesting. All retesting of cobas PCR media was performed within 12 months of the sample collection date in line with the manufacturer’s recommendations. NG culture data from bacteriological investigations were also available via routine testing for a limited number of samples. Routine media included blood agar, CHOC, and GC Lect (Oxoid; PathWest Media). Samples were cultured at 37°C in 5% CO2 for 48 hours. All NG isolates recovered from culture were identified using BD BBL Oxidase (Becton, Dickinson and Company, USA), MALDI Biotyper (Bruker Daltonik, GmbH), and VITEK 2 NH card (bioMérieux, France). Culture swabs were collected either at the time of sample collection for molecular testing, or subsequently within 12 days of the NG screening result. Susceptibility to ciprofloxacin was performed on all isolates using ETEST Ciprofloxacin (ETEST; bioMérieux, Marcy-l’Etoile, France) according to the instructions for use and interpreted using the Clinical and Laboratory Standards Institute (CLSI) guidelines (22). Culture results are described in the Supplementary Material.

### NG supplemental testing

As per Australian Public Health Laboratory Network (PHLN) guidelines for the use and interpretation of nucleic acid tests for the detection of NG, all samples were subjected to confirmatory testing (5, 8). For this study, all results (screen and confirmatory) were interpreted as per the assay kit inserts. Note that the RP-GC utilizes both *opa* and *porA* for NG detection and that samples, irrespective of the sample site, are considered positive for NG if either the *opa* or *porA* targets provide positive results.

In addition to RP-GC, we also conducted a pilot study using Xpert as an additional supplemental test for the c6800. For this testing, we performed Xpert for all samples that did not confirm using the above c6800/MP96-RP-GC workflow. We also applied Xpert to any extragenital samples that were confirmed by the MP96-RP-GC but were only positive by one RP-GC NG target (*opa* or *porA* positive; single confirmatory target positive) noting that pharyngeal samples are particularly prone to producing false-positive results in NG NAAT methods (5, 8). Xpert testing was recorded as detected or not detected according to the manufacturer’s instructions. All results for all samples are detailed in the Supplementary Material.

### Detection limit studies

The analytical sensitivity of c4800, c6800, c4800-RP-GC, MP96-RP-GC, and Xpert for the detection of NG was compared. In brief, a 10-fold dilution series of a quantified culture of *N. gonorrhoeae* ATCC 49226 (American Type Culture Collection, Manassas, VA, USA) was diluted with a matrix consisting of pooled NG-negative oropharyngeal specimens in cobas PCR media (*n* = 40). The dilutions covered the range of 1.00E+06 to 1.00E-01 colony forming units per milliliter (CFU/mL). The standards were tested in triplicate using c6800 and a quantitative standard curve was prepared from the mean cycle of quantitation (*C_q_*) at each dilution. Dilutions over the range of 1.00E+04 to 0.1 CFU/mL were also tested in triplicate for each assay (except Xpert due to cost implications). Results are expressed as log_10_ CFU/mL or CFU/mL, with the latter rounded to the nearest whole CFU. Results were also interpreted following the NG supplemental testing criteria above. The results are shown in the Supplementary Material.

### Data analysis

Two-tailed *P*-values from Fisher’s exact test are reported for all comparative analyses, except the agreement between the reported *gyrA* status and susceptibility testing where overall agreement is reported.

## RESULTS

### Supplementary material

The number of sample types, qualitative and quantitative results, including *C_q_* values and culture results for all 450 samples tested are shown in the Supplementary Material. All results, tables, and statistical analyses presented in this study have been interpreted from the data in the Supplementary Material.

### Evaluation of cobas screening results compared to RP-GC and Xpert

A summary of the screening results and the NG confirmatory rates tested with c4800, c6800, c4800-RP-GC, MP96-RP-GC, and Xpert is shown in Table 1. From 300 NG-positive archival samples, c4800 returned 298 NG-positive results. The two (previously c4800 positive) samples providing negative results in the c4800 in this retrospective testing (sample S50, oropharyngeal and sample S224, vagina; see Supplementary Material) tested positive with c6800 (*C_q_* values = 37.18 and 36.64, respectively) and quantified to 4 and 5 CFU/mL respectively, indicating detection at the reported lower limit of detection for c6800 (1.0 CFU/mL). Both samples confirmed with *opa* and *porA* using MP96-RP-GC; however, sample S50 was *opa* positive and *porA* negative, and sample S224 was negative for both *opa* and *porA* using c4800-RP-GC. By contrast, the c6800 returned 293 NG-positive results, with seven (previously c4800 positive) samples providing c6800 negative results (samples numbers were S5, S23, S48, S74, S110, S124, and S180). These samples were all oropharyngeal samples with c4800 *C_q_* values ranging from 31.8 to 38.4 cycles. All were *opa* and *porA* negative for both c4800-RP-GC and MP96-RP-GC. In addition, all were c4800-RP-GC *opa* and *porA* negative at the time of the initial c4800 routine screening (data not shown). These seven discrepant samples were tested with Xpert to investigate. All were reported as not detected with Xpert; however, samples S5, S23, and S48 (all from the same patient) were positive for the NG4 Xpert target only. The NG4 *C_q_* values were 37.5, 32.7, and 33.7 cycles respectively. For interest, we also show the results for *Chlamydia trachomatis* (CT) for all samples, with 19.0% (57/300) and 22.0% (66/300) reporting a CT-positive result for c4800 and c6800, respectively. Both c4800 and c6800 returned 150 negative results for all NG-negative archival samples.

**TABLE 1:** Summary of the screening and NG confirmatory results for all samples tested with c4800, c6800, c4800-RP-GC, MP96-RP-GC, and Xpert

Sample type	Total^*a*^	c4800 results	c4800-RP-GC results			c6800 results	MP96-RP-GC results			Xpert testing results	
		NG positive^*b*^	NG confirmed (%)^*c*^		NG not confirmed (%)^*d*^	NG positive^*e*^	NG confirmed (%)^*c*^		NG not confirmed (%)^*d*^	Xpert positive (%)	Xpert negative (%)
			Dual target	Single target			Dual target	Single target			
Urine	139	86	83 (96.5)	0	3 (3.5)	86	86 (100)	0	0	–^*h*^	–
Vaginal	37	27	27 (100)	0	0	28	28 (100)	0	0		–
Urethral	36	26	25 (96.2)	0	1 (3.8)	26	26 (100)	0	0	–	–
Endocervical	18	13	13 (100)	0	0	13	13 (100)	0	0	–	–
Total urogenital	230	152	148 (97.4)	0	4 (2.6)	153	153 (100)	0	0		
Oropharyngeal	133	88	58 (65.9)	13 (14.8)	17 (19.3)	82	70 (85.4)	4 (4.9)	8 (9.7)	4 (4.8)	8 (9.7)
Anorectal	85	56	54 (96.4)	0	2 (3.6)	56	55 (98.2)	0	1 (1.8)	0	1 (1.8)^*g*^
Joint fluid	1	1	1 (100)	0	0	1	1 (100)	0	0	–	–
Eye	1	1	0	0	1 (100)	1	1 (100)	0	0	–	–
Total extragenital	220	146	113 (77.4)	13 (8.9)	20 (13.7)	140	127 (90.7)	4 (2.9)	9 (6.4)	4 (2.9)	9 (6.4)
Sample total	450	298	261 (87.6)	13 (4.4)	24 (8.0)	293	280 (95.6)	4 (1.3)	9 (3.1)	4 (1.4)	9 (3.1)

^*a*^ Number of c4800 NG-positive (*n* = 300) and NG-negative (*n* = 150) samples collected for this study.

^*b*^ Based on c4800 screening.

^*c*^ Based on RP-GC testing; dual target, samples positive for *opa* and *porA*; single target, samples positive for *opa* or *porA.*

^*d*^ Based on RP-GC testing, samples negative for *opa* and *porA.*

^*e*^ Based on c6800 screening.

^*f*^ Based on Xpert testing according to the instructions for use.

^*g*^ Xpert sample adequacy control failed and the final Xpert NG result reported as invalid (NG2 and NG4 targets were negative).

^*h*^ -, refers to none tested.

### Comparison NG confirmatory rates

Table 1 shows the NG confirmatory rates according to sample type for the samples that tested positive for c4800 and c6800. The urogenital NG confirmatory rate for c4800-RP-GC was 97.4% (148/152) compared to MP96-RP-GC of 100% (153/153). This improvement was not statistically significant (*P* = 0.0605). The extragenital NG confirmatory rate was higher for MP96-RP-GC at 93.6% (131/140), compared to 86.3% (126/146) for c4800-RP-GC; however, the difference was not statistically significant (*P* = 0.6677). The oropharyngeal sample NG confirmatory rates were 90.2% (74/82) for MP96-RP-GC compared to 80.7% (71/88) for c4800-RP-GC, albeit again not statistically significant (*P* = 0.6520). The increase in NG confirmatory rates for MP96-RP-GC was primarily caused by improved detection of *porA* from MP96 nucleic acids compared to c4800 nucleic acids. Combining urogenital and extragenital samples, the confirmatory rate for MP96-RP-GC was 96.9% (284/293) compared to 91.9% (274/298) for c4800-RP-GC (*P* = 0.6796). In all, 13 extragenital samples were c6800 positive that were single target positive or did not confirm using MP96-RP-GC. These were tested with Xpert to investigate and four were positive (S31, S144, S248, and S287). The remaining nine samples were Xpert negative (all were negative for the Xpert NG2 and NG4 targets), with one sample (S42) returning an invalid result due to failure of the sample adequacy control. Combining all confirmed extragenital results from c6800, MP96-RP-GC, and Xpert, the final extragenital confirmatory rate was 96.4% (135/140) with a total overall confirmatory rate of 98.3% (288/293).

### Comparison of c4800-RP-GC and MP96-RP-GC *gyrA* results with ETEST results

Table 2 shows c4800-RP-GC and MP96-RP-GC *gyrA* results compared to ETEST for isolates recovered from culture (*n* = 63). From these isolates, 58.7% (37/63) were ETEST susceptible and 41.3% (26/63) were non-susceptible. The qualitative ETEST results closely correlate with the reported percentage for c4800-RP-GC and MP96-RP-GC according to the *gyrA* status, with MP96-RP-GC closest at 52.9% (155/293) for *gyrA* S91 and 39.2% (115/293) for *gyrA* S91F. The number of samples reporting a conclusive *gyrA* result for urogenital samples tested with MP96-RP-GC was 96.1% (147/153) compared to 90.8% (138/152) for c4800-RP-GC. This improvement using the MP96 extraction was not statistically significant (*P* = 0.0682). Fewer *gyrA* indeterminate results were observed for MP96-RP-GC (*n* = 6, 3.9%) compared to c4800-RP-GC (*n* = 14, 9.2%). The number of samples reporting a conclusive *gyrA* result for extragenital samples tested with MP96-RP-GC was 87.9% (123/140) compared to 73.3% (107/146) for c4800-RP-GC. This improvement was statistically significant (*P* = 0.0027). Fewer *gyrA* indeterminate results were observed for MP96-RP-GC (*n* = 17, 12.1%) compared to c4800-RP-GC (*n* = 39, 26.7%). Overall, the number of samples reporting a conclusive *gyrA* result for all samples was 92.2% (270/293) for MP96-RP-GC compared to 82.2% (245/298) for c4800-RP-GC. The improvement was statistically significant (*P* = 0.0003).

**TABLE 2:** Summary of the c4800, c6800, c4800-RP-GC *gyrA,* and MP96-RP-GC *gyrA* results compared ciprofloxacin ETEST results from recovered isolates

Sample type	c4800 results	c4800-RP-GC results			c6800 results	MP96-RP-GC results			Ciprofloxacin ETEST results from isolates			
	NG positive^*b*^	*gyrA* S91 (%)^*c*^	*gyrA* S91F (%)^*c*^	*gyrA* ind. (%)^*c*^	NG positive^*d*^	*gyrA* S91 (%)^*c*^	*gyrA* S91F (%)^*c*^	*gyrA* ind (%)^*c*^	No. isolates	S (%)^*e*^	R/NS (%)^*e*^	Concordance (%)^*f*^
Urine	86	51 (59.3)	27 (31.4)	8 (9.3)	86	55 (64.0)	27 (31.4)	4 (4.7)	11	8 (72.7)	3 (27.3)	100.0
Vaginal	27	15 (55.6)	9 (33.3)	3 (11.1)	28	17 (60.7)	9 (32.1)	2 (7.1)	6	3 (50.0)	3 (50.0)	100.0
Urethral	26	15 (57.7)	10 (38.5)	1 (3.8)	26	15 (57.7)	11 (42.3)	0	18	10 (55.6)	8 (44.4)	94.4^*g*^
Endocervical	13	7 (53.8)	4 (30.8)	2 (15.4)	13	7 (53.8)	6 (46.2)	0	5	4 (80.0)	1 (20.0)	100.0
Total urogenital	152	88 (57.9)	50 (32.9)	14 (9.2)	153	94 (61.4)	53 (34.6)	6 (3.9)	40	25 (62.5)	15 (37.5)	97.5^*g*^
Oropharyngeal	88	25 (28.4)	30 (34.1)	33 (37.5)	82	29 (35.4)	37 (45.1)	16 (19.5)	10	4 (40.0)	6 (60.0)	100.0
Anorectal	56	28 (50.0)	23 (41.1)	5 (8.9)	56	31 (55.4)	24 (42.9)	1 (1.8)	13	8 (61.5)	5 (38.5)	100.0
Joint fluid	1	0	1 (100.0)	0	1	0	1 (100.0)	0	0	-	-	-
Eye	1	0	0	1 (100.0)	1	1 (100.0)	0	0	0	-	-	-
Total extragenital	146	53 (36.3)	54 (37.0)	39 (26.7)	140	61 (43.6)	62 (44.3)	17 (12.1)	23	12 (52.2)	11 (47.8)	100.0
Sample total^*a*^	298	141 (47.3)	104 (34.9)	53 (17.8)	293	155 (52.9)	115 (39.2)	23 (7.8)	63	37 (58.7)	26 (41.3)	98.4^*g*^

^*a*^ From all positive samples collected for this study (*n* = 300).

^*b*^ Number of NG-positive samples from c4800 testing.

^*c*^ *gyrA* status based on RP-GC testing (*gyrA* S91, wild type associated with ciprofloxacin sensitivity; *gyrA* S91F, mutation associated with ciprofloxacin resistance; *gyrA* ind., *gyrA* status indeterminate).

^*d*^ Number of NG-positive samples from c6800 testing.

^*e*^ Number of isolates recovered from culture and ciprofloxacin ETEST results; S = sensitive, R/NS = resistant/non-susceptible.

^*f*^ Percent concordance comparing *gyrA* status and ciprofloxacin ETEST results.

^*g*^ Urethral sample S176 was *gyrA* S91F positive with a ciprofloxacin ETEST minimum inhibitory concentration of 0.064 (S). Isolate recovered from culture also tested as *gyrA* S91F positive.

The RP-GC *gyrA* testing results were concordant with available culture data for 61/62 samples. An isolate recovered from a urethral swab at the same day and time of sample collection for molecular screening (sample S176) was discordant with the *gyrA* status for both c4800-RP-GC and MP96-RP-GC compared to susceptibility testing. The sample was determined to be resistant by RP-GC but ciprofloxacin susceptible by culture. This sample was a high titer sample (5.3 log_10_ CFU/mL) with an observed *gyrA* S91F *C_q_* of 16.7 with a nominal *gyrA* S91 *C_q_* of 28.4. Other samples from the same patient on the same day (urine sample S175 and oropharyngeal sample S177) were also *gyrA* S91F positive for both c4800-RP-GC and MP96-RP-GC. The isolate demonstrated a ciprofloxacin ETEST MIC result of 0.064, which is considered sensitive according to CLSI guidelines. The isolate was recovered from −80°C glycerol storage and repeat ciprofloxacin ETEST was performed with consistent results (MIC 0.064). The original sample and the recovered isolate were retested with MP96-RP-GC. In both cases, the *gyrA* S91F mutation was detected. The reported *gyrA* status correlated with the susceptibility result for all other isolates (*n* = 62; 26 non-susceptible and 36 susceptible). The overall agreement between the reported *gyrA* status and susceptibility testing was 98.4% (confidence interval estimate at 95% confidence of 91.5%–99.7%).

### Analysis of positive c4800 and c6800 samples that were RP-GC and Xpert negative

Table 3 shows the qualitative and quantitative results for eight samples that were c4800 and c6800 positive that were negative for c4800-RP-GC, MP96-RP-GC, and Xpert targets. From the Supplementary Material, Sample S115 was culture negative, and all other samples were not cultured. Samples S42 and S115 demonstrated quantitative NG results of <10 CFU/mL. Samples S192 and S257 demonstrated quantitative NG results of 10–100 CFU/mL. Samples S35, S214, and S223 demonstrated quantitative NG results of 100–1000 CFU/mL. Sample S205 was the highest titer demonstrating a quantitative NG result of 2,459 CFU/mL. Sample S42 (anorectal) demonstrated a late Xpert sample processing control (SPC) *C_q_* value (40.5), possibly indicating the presence of interfering substances. This sample also failed to amplify the sample adequacy control (SAC), which may be a consequence of interfering substances or lack of exogenous human DNA. All other samples demonstrated nominal internal control (IC) and SPC *C_q_* values when compared to the results for oropharyngeal samples. The average *C_q_* and standard deviation of the MP96-RP-GC IC for all c6800 positive oropharyngeal samples was 24.41 ± 0.66 *C_q_*. Similarly, anorectal samples were 24.62 ± 0.66 *C_q_* and all urogenital samples were 24.50 ± 0.66 *C_q_*. Excluding the seven oropharyngeal samples presented in Table 3, all other oropharyngeal samples with a quantitative NG result >1 CFU/mL (*n* = 74) confirmed with MP96-RP-GC (*opa* and *porA*) (*n* = 70, 94.6%), with the remaining confirmed results (*n* = 4) demonstrating a positive result with a single confirmatory target (*opa*), with three samples (S31, S144, S287) positive for Xpert.

**TABLE 3:** Summary of positive c4800 and c6800 samples that were negative for all RP-GC and Xpert targets

Sample	Patient	Sample type	c4800 results	c4800-RP-GC results^*b*^		c6800 results		MP96-RP-GC results^*d*^		Xpert results^*e*^		
			NG *C_q_ ^a^*	*opa* and *porA*	IC *C_q_^a^*	NG *C_q_^a^*	NG CFU (log_10_)/mL^*c*^	*opa* and *porA*	IC *C_q_^a^*	NG2 and NG4	SAC *C_q_^a^*	SPC *C_q_^a^*
S35	P23	Oropharyngeal	35.7	Negative	24.62	30.27	381 (2.58)	Negative	24.62	Negative	22.9	30.8
S42	P27	Anorectal	38.4	Negative	25.62	38.50	2 (0.19)	Negative	25.62	Negative^*f*^	Failed^*f*^	40.5
S115	P86	Oropharyngeal	37.7	Negative	24.45	37.55	3 (0.47)	Negative	24.45	Negative	28.4	32.1
S192	P142	Oropharyngeal	38.2	Negative	24.69	35.11	15 (1.18)	Negative	24.69	Negative	29.7	31.6
S205	P153	Oropharyngeal	33.6	Negative	24.14	27.48	2,459 (3.39)	Negative	24.14	Negative	21.2	30.5
S214	P153	Oropharyngeal	35.6	Negative	24.54	29.00	891 (2.95)	Negative	24.54	Negative	24.1	30.7
S223	P153	Oropharyngeal	35.9	Negative	24.49	31.50	167 (2.22)	Negative	24.49	Negative	23.3	31.6
S257	P181	Oropharyngeal	36.7	Negative	24.25	33.76	37 (1.57)	Negative	24.25	Negative	24.1	31.5

^*a*^ *C_q_*, cycle of quantitation.

^*b*^ RP-GC results from c4800 nucleic acids (IC, internal control).

^*c*^ From NG standard curve (Supplementary Material); CFU, colony forming units.

^*d*^ RP-GC results from MP96 nucleic acids (IC, internal control).

^*e*^ Xpert results from cobas PCR media (NG2 and NG4, chromosomal NG targets; SAC, sample adequacy control; SPC, sample process control).

^*f*^ Xpert sample adequacy control failed and the final Xpert NG result was reported as invalid (NG2 and NG4 targets were negative).

### Analysis of the detection limit results using quantitative analysis

The results for the c6800 NG standard curve and detection limit studies comparing c4800, c6800, c4800-RP-GC, MP96-RP-GC, and Xpert are shown in the Supplementary Material. The NG standard curve of NG ATCC 49226 was log-linear with an *R*^2^ value of 0.9994. The regression formula for calculating the log_10_ CFU/mL value for any given sample *C_q_* value was *y* = −0.*2904x* + 11.371. The standard curve covered the quantitative range typically observed for all clinical samples (Supplementary Material). All three replicates at 1 CFU/mL were positive which correlates with the reported lower limit of detection for c6800 according to the manufacturer. The detection limit studies showed c6800 to be 2 log dilutions more sensitive than c4800 (all three replicates positive), with c6800 at 1 CFU/mL compared to 100 CFU/mL for c4800. The MP96-RP-GC showed to be 1 log dilution more sensitive than c4800-RP-GC (all three replicates *opa* and *porA* positive), with MP96-RP-GC at 100 CFU/mL and c4800-RP-GC at 1000 CFU/mL. Overall, *opa* was more sensitive than *porA* for c4800-RP-GC and MP96-RP-GC. Xpert was 1 log dilution less sensitive than c6800, with Xpert at 10 CFU/mL compared to c6800 at 1 CFU/mL. The limits of detection for each commercial assay are similar to the package insert claims.

### Routine culture

All culture results are shown in the Supplementary Material. Culture requested at the time of collection represented 25.7% (77/300) of the total compared to PCR-only requests at 74.3% (223/300) of the total. Subsequent culture requests 1–11 days after the screening swab was collected accounted for 6.7% (20/300) of the total. The total recovery rate from culture was 70.0% (64/97) with 63.6% (49/77) recovered from the time of collection and 75% (15/20) from subsequent culture requests.

## DISCUSSION

Our results conclusively show an increase in the overall NG confirmatory rate for MP96-derived nucleic acids compared to c4800 nucleic acids, with oropharyngeal samples as the key point of difference. Although the increase in overall NG confirmatory rate was not statistically significant, we demonstrated a significant increase (*P* = 0.0003) in reporting a valid *gyrA* status, with fewer indeterminate results for MP96-RP-GC compared to c4800-RP-GC. We conclude that both increases are related to the improved sensitivity of MP96-RP-GC as evidenced in the detection limit studies. The increase in sensitivity may be due to higher nucleic acid yield or more optimal PCR performance. Regardless, the improvement in MP96-RP-GC test performance is welcomed for a number of reasons: (i) the c6800 has no retrievable nucleic acids for supplemental testing; therefore, separate extraction is mandatory, (ii) a more sensitive NG confirmatory test is beneficial, particularly for oropharyngeal samples, with the improved analytical sensitivity of c6800 compared to c4800, and (iii) additional sensitive or resistant *gyrA* results further improve the clinical utility of the RP-GC assay.

We demonstrate that the c6800 followed by the MP96-RP-GC testing is an improved supplemental testing method compared to c4800-RP-GC with a 100% confirmatory rate for urogenital samples and 93.6% for extragenital samples. As with our previous study and others (7, 11), we conclude that supplemental testing is not required for c6800 for urogenital samples, but oropharyngeal samples should undergo secondary testing, in line with current Australian and UK guidelines (5, 10). At the time of our previous study, the US version of the c6800 CTNG test did not have an oropharyngeal or anorectal claim, whereas the CE/IVD marked kits approved for use in Australia and the UK were approved for use for these sample types. Recent versions are now harmonized and include both oropharyngeal and anorectal samples. Despite this, our data show that there are still some unexplained occurrences of oropharyngeal positive samples which cannot be confirmed using other NG-NAATS. This has been previously demonstrated in our earlier study and other investigations (7, 11). Another evaluation has shown discrepant c6800 positive and c4800 negative results for oropharyngeal samples; however an independent supplemental NAAT was not used to investigate these discordant results (6).

Based on our data, it is unlikely that the discrepancies are due to the differences in the analytical sensitivity of c6800 compared to Xpert. The claimed sensitivity of c6800 irrespective of sample type is 1 CFU/mL which was confirmed experimentally in this study. This same detection limit has been confirmed by others (6). Furthermore, the claimed sensitivity of Xpert with a pooled pharyngeal swab matrix sensitivity is 6.4 CFU/mL. Again, this was confirmed experimentally in this study, with Xpert 10-fold less sensitive than c6800. In both cases, we used the same ATCC strain used by the manufacturers. Finally, we developed an in-house method for the quantification of NG using a pooled oropharyngeal swab matrix. The standard curve was applied to c6800 for the limit of detection studies and all clinical samples tested. Although the standard was prepared using an oropharyngeal matrix and then applied to non-oropharyngeal samples, the manufacturer has stated a limit of 1 CFU/mL for all sample types tested, with the ability to detect *N. gonorrhoeae* strains below 1 CFU/mL. Therefore, we consider our quantitative approach valid and worst-case scenario in terms of encountering interfering substances and other non-gonococcal *Neisseria* species in the oropharyngeal matrix. Based on the premise that c6800 and MP96-RP-CG detected and confirmed all urogenital samples and 93.6% of extragenital samples greater than 2 CFU/mL, of which three were positive with Xpert, we conclude that the majority of samples presented in Table 3 should have at least flagged positive for a single target with an alternative NAAT based on the NG load. Furthermore, we expected the samples with quantitative values between 100 and 2500 CFU/mL to confirm with MP96-RP-GC and/or detected with Xpert. We acknowledge that cobas PCR media is not a validated reagent for Xpert. However, Xpert also uses a similar guanidinium chloride-based media for urine. Therefore, we consider cobas PCR media compatible with Xpert and have shown that cobas PCR media performs well for oropharyngeal samples.

In terms of improved specificity, we have shown that a previously documented case of *N. macacae* providing false-positive results with c4800 does not cross-react with the later generation c6800 assay (23). From 300 c4800 NG-positive samples collected from initial screening, we observed seven samples that were c6800 negative. Not surprisingly, all were oropharyngeal samples. By contrast, an earlier Roche-funded study comparing c4800 and c6800 did not encounter any c4800-positive oropharyngeal samples with c6800-negative results (6). Our discordant cases were c4800-RP-GC, MP96-RP-GC, and Xpert negative. Three were positive for the Xpert NG4 target; however, this target has been shown to cross-react with non-gonococcal *Neisseria* species (24, 25). These discrepancies may be examples of the aforementioned c4800 non-specificity, but we were unable to recover an isolate from culture to confirm. We conclude some improvement in specificity with the c6800 test compared to c4800 from this observation alone. We also demonstrate improvement in the oropharyngeal NG confirmatory rate using MP96-RP-GC combined with Xpert. Following this approach, the number of oropharyngeal samples was unable to be confirmed accounting for 8.5% of the total (7/82). By contrast, in a recent report using c6800 as the screening test followed by a supplemental test targeting the *pilin inversion* (*pivNG*) gene using the cobas omni channel, the investigators were unable to confirm 23.7% (27/114) c6800-positive oropharyngeal samples with *pivNG* and Xpert combined, despite reporting equal detection limits in the supplemental material for *pivNG* compared to c6800 (albeit with commercial control material in either cobas PCR media or water) (11). The investigators state sample volumes of 400 µL for the omni channel *pivNG* and 300 µL for Xpert. By contrast, we use 200 µL for MP96 and 1 mL for Xpert. We note that the first version of Xpert CT/NG (301–0234, Rev. B, January 2013) and subsequent versions available to our laboratory have consistently stated a sample input volume of 1 mL. Following our approach, an overall confirmation rate of 98.3% was achieved with supplemental assays analytically less sensitive compared to c6800 when tested with pooled oropharyngeal matrix. The detection limits we observed are also similar to those reported by the manufacturer, using an oropharyngeal matrix. As a diagnostic strategy, we consider maximizing the NG confirmatory rate and reporting an AMR marker as a more advantageous trade-off than reduced sample handling and *pivNG* testing with the omni channel.

The RP-GC assay utilizes *PlexZyme* technology (26) to simultaneously detect *opa* and *porA* with *gyrA* S91 (wild type) or *gyrA* S91F (mutant) in a single PCR. We also observed an improvement in the *gyrA* status reporting for RP-GC using MP96 nucleic acids compared to c4800 nucleic acids, with fewer *gyrA* indeterminate results for MP96-RP-GC. Given that in 2021, 47% of all reported NG isolates are ciprofloxacin susceptible in Australia and even higher in metropolitan (63.6%) and remote (96.4%) areas of Western Australia (27), a large proportion could be potentially treated with ciprofloxacin, despite being unsuitable for empirical treatment on the basis of being well above the WHO 5% resistance threshold (28). Current and historical recommended first-line antimicrobials in many countries for NG exceed the 5% resistance threshold (13). Given that greater than 5% resistance to ceftriaxone could be reached by 2030 based on mathematical models (29), improved strategies for continued antimicrobial stewardship and new diagnostic tests to allow resistance-guided therapy for the 50–70% of isolates susceptible to ciprofloxacin are needed (15, 30). When compared with the corresponding bacterial culture results, the positivity of the *gyrA* S91 or *gyrA* S91F at the time of NG supplemental testing, correctly predicted NG susceptibility to ciprofloxacin in 62 of 63 samples (98.4%). The discrepant sample was further investigated. The original sample was retested with MP96-RP-GC with consistent results. Subsequent subculture from storage and retesting isolate with ETEST and MP96-RP-GC revealed consistent results. We confirmed the *gyrA* S91F-resistant mutation for the isolate with an ETEST MIC of 0.064 (sensitive) according to CLSI criteria (≤0.06 reported as sensitive). It is unlikely that the discrepancy is due to different strains and the reason for the ciprofloxacin susceptible result is unknown. Nevertheless, we do not see a “major error” (false resistance) as a problem as this simply means ciprofloxacin would be ruled out for this patient. A “very major error” (false susceptibility) where ciprofloxacin was inappropriately ruled in would have otherwise been a problem.

Our study has limitations. Although our sample size for positive samples collected over a year of testing was sufficient (*n* = 300), the number of negative samples tested was half (*n* = 150) and not reflective of the positive and negative prevalence of the population tested. Determining c6800 performance and NG confirmation with MP96-RP-GC compared to c4800 using a much larger negative sample set screened with c4800 would be ideal to determine the true number of c6800-positive samples compared to c4800-negative samples, given the increase in the analytical sensitivity of c6800 compared to c4800. This would be of value for oropharyngeal samples in particular, given we may encounter more NG-positive samples that are RP-GC and Xpert negative. Similarly, a larger number of ciprofloxacin susceptibility results compared to *gyrA* would have been ideal; although ETEST concordance with the *gyrA* status was high, only 22% (63/298) of c6800-positive samples had isolates available for ciprofloxacin susceptibility testing. It should also be noted that our statistical approaches considered all patient samples to be independent and did not consider the broader infected patient status; however, this was considered necessary for the study given the objectives focused on maximizing detection of NG and AMR within individual samples and not collectively for all samples from an individual patient. This is further supported by the fact that as per routine practice, all samples testing positive for NG from any site are sent for NG confirmation and this is irrespective of results of any other sites from the same patient. Finally, we have already acknowledged that cobas PCR media is not a manufacturer-validated reagent for Xpert; however, based on this study and our previous work (7), cobas PCR media has no discernible impact on Xpert test performance. Although all samples in cobas PCR media were retested within 12 months (based on the confirmed stability studies of the manufacturer), similar stability for Xpert is assumed.

In conclusion, this is the first study to show the clinical performance of the RP-GC assay in conjunction with c4800 and c6800 CT/NG. We demonstrate exceptionally high NG confirmatory rates for urogenital and extragenital samples combining RP-GC with MP96 extraction. In addition, we also show comparable sensitivity of *gyrA* detection compared to NG confirmation while confirming the reliability of *gyrA* to predict ciprofloxacin susceptibility (19). We also highlight the ongoing need for supplemental testing for oropharyngeal samples and the importance of genotypic AMR detection, as a progressive step toward specific individualized treatment to reduce the growing state of AMR in gonococcus.

## SUPPLEMENTAL MATERIAL

NG confirmation supplemental material jcm.01039-23-s0001.xlsx Excel NG supplemental file.

ASM does not own the copyrights to Supplemental Material that may be linked to, or accessed through, an article. The authors have granted ASM a non-exclusive, world-wide license to publish the Supplemental Material files. Please contact the corresponding author directly for reuse.

## References

[1] Rowley J, Vander Hoorn S, Korenromp E, Low N, Unemo M, Abu-Raddad LJ, Chico RM, Smolak A, Newman L, Gottlieb S, Thwin SS, Broutet N, Taylor MM. 2019. Chlamydia, gonorrhoea, trichomoniasis and syphilis: global prevalence and incidence estimates, 2016. Bull World Health Organ 97:548–562P. doi:10.2471/BLT.18.228486 31384073 PMC6653813

[2] Meyer T, Buder S. 2020. The laboratory diagnosis of Neisseria gonorrhoeae: current testing and future demands. Pathogens 9:91. doi:10.3390/pathogens9020091 32024032 PMC7169389

[3] Upton A, Bromhead C, Whiley DM. 2013. Neisseria gonorrhoeae false-positive result obtained from a pharyngeal swab by using the roche cobas 4800 CT/NG assay in New Zealand in 2012. J Clin Microbiol 51:1609–1610. doi:10.1128/JCM.00485-13 23486711 PMC3647902

[4] Field N, Clifton S, Alexander S, Ison CA, Hughes G, Beddows S, Tanton C, Soldan K, Coelho da Silva F, Mercer CH, Wellings K, Johnson AM, Sonnenberg P. 2015. Confirmatory assays are essential when using molecular testing for Neisseria gonorrhoeae in low-prevalence settings: insights from the third national survey of sexual attitudes and lifestyles (Natsal-3). Sex Transm Infect 91:338–341. doi:10.1136/sextrans-2014-051850 25512673 PMC4518812

[5] Whiley DM, Lahra MM, National Neisseria Network. 2015. Review of 2005 public health laboratory network Neisseria gonorrhoeae nucleic acid amplification tests guidelines. Commun Dis Intell Q Rep 39:E42–E45. 26063097 10.33321/cdi.2015.39.6

[6] Marlowe EM, Hardy D, Krevolin M, Gohl P, Bertram A, Arcenas R, Seiverth B, Schneider T, Liesenfeld O. 2017. High-throughput testing of urogenital and extragenital specimens for detection of Chlamydia trachomatis and Neisseria gonorrhoeae with Cobas® CT/NG. Eur J Microbiol Immunol 7:176–186. doi:10.1556/1886.2017.00018 PMC5632745 29034107

[7] Pryce TM, Hiew VJ, Haygarth EJ, Whiley DM. 2021. Second- and third-generation commercial Neisseria gonorrhoeae screening assays and the ongoing issues of false-positive results and confirmatory testing. Eur J Clin Microbiol Infect Dis 40:67–75. doi:10.1007/s10096-020-04004-5 32767178

[8] Smith DW, Tapsall JW, Lum G. 2005. Guidelines for the use and interpretation of nucleic acid detection tests for Neisseria gonorrhoeae in Australia: a position paper on behalf of the public health laboratory network. Commun Dis Intell Q Rep 29:358–365. 16465924 10.33321/cdi.2005.29.39

[9] Bromhead C, Miller A, Jones M, Whiley D. 2013. Comparison of the cobas 4800 CT/NG test with culture for detecting Neisseria gonorrhoeae in genital and nongenital specimens in a low-prevalence population in New Zealand. J Clin Microbiol 51:1505–1509. doi:10.1128/JCM.03223-12 23467604 PMC3647945

[10] Fifer H, Saunders J, Soni S, Sadiq ST, FitzGerald M. 2020. UK national guideline for the management of infection with Neisseria gonorrhoeae. Int J STD AIDS 31:4–15. doi:10.1177/0956462419886775 31870237

[11] Hopkins M, Arcenas R, Couto-Parada X, Lewinski M, Njoya M, Perinpanathan D, Sheriff R, Hansra A, Singh S. 2023. PivNG primers and probes set used in the cobas omni utility channel is a reliable supplemental test for detection of Neisseria gonorrhoeae in oropharyngeal, urogenital and rectal specimens collected in cobas PCR media. Sex Transm Infect 99:416–419. doi:10.1136/sextrans-2022-055576 37116988 PMC10447364

[12] Hook EW, Van Der Pol B. 2013. Evolving gonococcal antimicrobial resistance: research priorities and implications for management. Sex Transm Infect 89 Suppl 4:iv60–iv62. doi:10.1136/sextrans-2013-051021 24243882

[13] Unemo M, Lahra MM, Escher M, Eremin S, Cole MJ, Galarza P, Ndowa F, Martin I, Dillon J-A, Galas M, Ramon-Pardo P, Weinstock H, Wi T. 2021. WHO global antimicrobial resistance surveillance for Neisseria gonorrhoeae 2017-18: a retrospective observational study. Lancet Microbe 2:e627–e636. doi:10.1016/S2666-5247(21)00171-3 35544082

[14] Mohammed H, Ison CA, Obi C, Chisholm S, Cole M, Quaye N, Hughes G, GRASP Collaborative Group. 2015. Frequency and correlates of culture-positive infection with Neisseria gonorrhoeae in England: a review of sentinel surveillance data. Sex Transm Infect 91:287–293. doi:10.1136/sextrans-2014-051756 25352692

[15] Pond MJ, Hall CL, Miari VF, Cole M, Laing KG, Jagatia H, Harding-Esch E, Monahan IM, Planche T, Hinds J, Ison CA, Chisholm S, Butcher PD, Sadiq ST. 2016. Accurate detection of Neisseria gonorrhoeae ciprofloxacin susceptibility directly from genital and extragenital clinical samples: towards genotype-guided antimicrobial therapy. J Antimicrob Chemother 71:897–902. doi:10.1093/jac/dkv432 26817487 PMC4790619

[16] Siedner MJ, Pandori M, Castro L, Barry P, Whittington WLH, Liska S, Klausner JD. 2007. Real-time PCR assay for detection of quinolone-resistant Neisseria gonorrhoeae in urine samples. J Clin Microbiol 45:1250–1254. doi:10.1128/JCM.01909-06 17267635 PMC1865802

[17] Low N, Unemo M. 2016. Molecular tests for the detection of antimicrobial resistant Neisseria gonorrhoeae: when, where, and how to use? Curr Opin Infect Dis 29:45–51. doi:10.1097/QCO.0000000000000230 26658656

[18] Goire N, Lahra MM, Chen M, Donovan B, Fairley CK, Guy R, Kaldor J, Regan D, Ward J, Nissen MD, Sloots TP, Whiley DM. 2014. Molecular approaches to enhance surveillance of gonococcal antimicrobial resistance. Nat Rev Microbiol 12:223–229. doi:10.1038/nrmicro3217 24509781

[19] Buckley C, Trembizki E, Donovan B, Chen M, Freeman K, Guy R, Kundu R, Lahra MM, Regan DG, Smith H, Whiley DM, Investigators GS. 2016. A real-time PCR assay for direct characterization of the Neisseria gonorrhoeae GyrA 91 locus associated with ciprofloxacin susceptibility. J Antimicrob Chemother 71:353–356. doi:10.1093/jac/dkv366 26538505

[20] Allan-Blitz L-T, Humphries RM, Hemarajata P, Bhatti A, Pandori MW, Siedner MJ, Klausner JD. 2017. Implementation of a rapid genotypic assay to promote targeted ciprofloxacin therapy of Neisseria gonorrhoeae in a large health system. Clin Infect Dis 64:1268–1270. doi:10.1093/cid/ciw864 28034887 PMC5399946

[21] Spratt BG, Bowler LD, Zhang QY, Zhou J, Smith JM. 1992. Role of interspecies transfer of chromosomal genes in the evolution of penicillin resistance in pathogenic and commensal Neisseria species. J Mol Evol 34:115–125. doi:10.1007/BF00182388 1556747

[22] Performance Standards for Antimicrobial Susceptibility Testing. 2021. CLSI supplement M100 (ISBN 978-1-68440-104-8 [print]; ISBN 978-1-68440-105 [electronic]. 31st ed. Clinical and laboratory Standards Institute, USA.

[23] Pryce TM, Bromhead C, Whiley DM. 2023. A previously documented Neisseria macacae isolate providing a false-positive result with roche cobas 4800 CT/NG does not cross-react with the later generation cobas 6800 CT/NG assay. Eur J Clin Microbiol Infect Dis 42:121–123. doi:10.1007/s10096-022-04519-z 36372865

[24] Tabrizi SN, Unemo M, Golparian D, Twin J, Limnios AE, Lahra M, Guy R, TTANGO Investigators. 2013. Analytical evaluation of GeneXpert CT/NG, the first genetic point-of-care assay for simultaneous detection of Neisseria gonorrhoeae and chlamydia trachomatis. J Clin Microbiol 51:1945–1947. doi:10.1128/JCM.00806-13 23554203 PMC3716077

[25] Jacobsson S, Boiko I, Golparian D, Blondeel K, Kiarie J, Toskin I, Peeling RW, Unemo M. 2018. WHO laboratory validation of Xpert® CT/NG and Xpert® TV on the GeneXpert system verifies high performances. APMIS 126:907–912. doi:10.1111/apm.12902 30456870 PMC6488022

[26] Mokany E, Tan YL, Bone SM, Fuery CJ, Todd AV. 2013. MNAzyme qPCR with superior multiplexing capacity. Clin Chem 59:419–426. doi:10.1373/clinchem.2012.192930 23232065

[27] Lahra MM, Hogan TR, Armstrong BH. 2022. Australian gonococcal surveillance programme annual report, 2021. Commun Dis Intell (2018) 46:10.33321/cdi.2022.46.52. doi:10.33321/cdi.2022.46.52 35981810

[28] Wi T, Lahra MM, Ndowa F, Bala M, Dillon J-A, Ramon-Pardo P, Eremin SR, Bolan G, Unemo M. 2017. Antimicrobial resistance in Neisseria gonorrhoeae: global surveillance and a call for international collaborative action. PLoS Med 14:e1002344. doi:10.1371/journal.pmed.1002344 28686231 PMC5501266

[29] Riou J, Althaus CL, Allen H, Cole MJ, Grad YH, Heijne JCM, Unemo M, Low N. 2023. Projecting the development of antimicrobial resistance in Neisseria gonorrhoeae from antimicrobial surveillance data: a mathematical modelling study. BMC Infect Dis 23:252. doi:10.1186/s12879-023-08200-4 37081443 PMC10116452

[30] Marks M, Harding-Esch E. 2021. Antimicrobial resistance in gonorrhea: diagnostics to the rescue. Clin Infect Dis 73:304–305. doi:10.1093/cid/ciaa591 32766713

